# Oncology Organization and Oncologist Networks Under Medicare Advantage Plans

**DOI:** 10.1001/jamanetworkopen.2026.18507

**Published:** 2026-06-15

**Authors:** Xin Hu, Youngmin Kwon, Qinjin Fan, Kewei Sylvia Shi, Zhiyuan Zheng, Jingxuan Zhao, Joan L. Warren, K. Robin Yabroff, Zhanji Zhang, Xuesong Han, Changchuan Jiang

**Affiliations:** 1 Department of Radiation Oncology, School of Medicine, Emory University, Atlanta, Georgia; 2 Winship Cancer Institute, Emory University, Atlanta, Georgia; 3 Department of Health Policy and Management, Rollins School of Public Health, Emory University, Atlanta, Georgia; 4 Department of Health Policy, Vanderbilt University Medical Center, Nashville, Tennessee; 5 Department of Surveillance, Prevention, & Health Services Research, American Cancer Society, Atlanta, Georgia; 6 Department of Internal Medicine, University of Texas Southwestern Medical Center, Dallas

## Abstract

**Importance:**

More than half of Medicare beneficiaries are now enrolled in Medicare Advantage (MA) plans. These plans offer lower premiums and additional benefits compared with Traditional Medicare (TM) but commonly restrict organization and clinician networks, potentially limiting access to specialists, including oncologists. Little is known about the use of oncology networks in MA plans.

**Objective:**

To evaluate effective oncology network breadth under MA plans based on realized health care utilization.

**Design, Setting, and Participants:**

This cross-sectional study used linked 2016-2019 Surveillance, Epidemiology, and End Results (SEER)–Medicare data to characterize effective oncology networks among beneficiaries diagnosed with 8 common cancers. Network trends were examined separately for regular MA plans and special needs plans (SNPs) within SEER counties, and network breadth was compared by metropolitan status and plan type (health maintenance organization [HMO], local or regional preferred provider organization [PPO], point-of-service [POS] plan, or other). Data were analyzed between March 1, 2025, and April 1, 2026.

**Main Outcomes and Measures:**

Effective oncology network breadth was defined at the plan-county-year level as the share of oncology organizations and oncologists accessed by MA beneficiaries relative to oncology organizations and oncologists accessed by TM beneficiaries in the same county-year, measured separately for oncology organizations, medical or surgical oncologists, and radiation oncologists and more than 1 MA-recorded visit to a National Cancer Institute (NCI)–designated comprehensive cancer center.

**Results:**

A total of 807 580 MA beneficiaries (mean [SD] age, 70.4 [9.0] years; 420 662 males [52.1%]) were identified, representing 23 255 plan-year observations for regular MA plans and 17 716 for SNPs from 2016 through 2019. Across regular MA plans, beneficiaries accessed a mean (SD) of 12.0% (12.7%) of oncology organizations, 6.8% (9.6%) of medical or surgical oncologists, and 11.6% (13.8%) of radiation oncologists; across SNPs, beneficiaries accessed 12.4% (12.6%) of oncology organizations, 7.2% (8.6%) of medical or surgical oncologists, and 12.7% (15.5%) of radiation oncologists. MA-recorded visits to NCI-designated comprehensive cancer centers were observed in 25.7% (n = 5983 of 23255) of regular MA plans and 20.5% (n = 3624 of 17716) of SNPs. Effective oncology network breadth was narrower for HMOs; nonmetropolitan (vs metropolitan) counties and regional PPOs (vs other plan types) had lower likelihood of MA-recorded visits to NCI-designated comprehensive cancer centers.

**Conclusions and Relevance:**

In this cross-sectional study of MA plans in SEER regions from 2016 to 2019, effective oncology organization and oncologist networks were constrained. Restricted access to NCI-designated comprehensive cancer centers might limit access to innovative treatments and cancer outcomes, which warrants future research.

## Key Points

**Question:**

What is the effective oncology organization and oncologist network breadth in Medicare Advantage (MA) plans based on realized health care utilization?

**Findings:**

Using 2016-2019 Surveillance, Epidemiology, and End Results–Medicare data of 23 255 regular MA plan-years and 17 716 special needs plan-years for 807 580 MA enrollees, this cross-sectional study found that beneficiaries accessed approximately 12% of oncology organizations, 7% of medical or surgical oncologists, and 12% of radiation oncologists vs Traditional Medicare beneficiaries. Fewer than one-quarter of MA plans covered National Cancer Institute–designated comprehensive cancer centers, and effective networks were narrower in HMOs and nonmetropolitan areas.

**Meaning:**

These findings suggest constrained effective oncology networks may limit access to specialized cancer care; research on the potential implications of constrained networks for cancer outcomes is warranted.

## Teaser

This cross-sectional study uses SEER-Medicare data to examine the oncology organization and oncologist network breadth of Medicare Advantage plans for beneficiaries with cancer compared with those enrolled in Traditional Medicare.

## Introduction

Enrollment in Medicare Advantage (MA) plans, the privately administered alternatives to Traditional Medicare (TM), has grown rapidly and surpassed TM for the first time in 2023, now covering more than half of all beneficiaries.^1,2^ MA plans receive capitated payments from Medicare,^3^ which incentivizes care efficiency and allows plans to offer supplemental benefits, such as dental, vision, or transportation services not covered under TM. Furthermore, MA plans limit beneficiaries’ financial exposure through lower cost sharing and annual out-of-pocket maximums not available under TM.

However, capitated payments also create strong incentives for cost-containment strategies, such as restrictive organization and clinician networks.^4^ Restrictive networks are particularly consequential for patients with high-cost, high-complexity conditions, such as cancer. Patients with cancer increasingly require access to subspecialty expertise, novel therapeutics, and clinical trials,^5,6^ many of which are concentrated in high-volume academic and National Cancer Institute (NCI)–designated comprehensive cancer centers.^7,8^ Increasing multimorbidity among older adults with cancer and the different toxicity profiles of novel cancer therapies intensify the need for coordinated multidisciplinary care.^9^ Although MA plans may rely on restricted networks to facilitate care coordination through contracting with high-quality organizations and clinicians, such restrictions may simultaneously impede access to advanced oncology services and adversely affect cancer outcomes.^10,11^

Narrow organization and clinician networks under MA plans have been documented across multiple specialties.^12^ Existing studies primarily quantified nominal networks^13^ derived from plan directories and defined a plan as narrow when it included fewer than 25% to 30% of local physicians. These directory-based analyses suggest that average MA plans include fewer than half of physicians nationwide^14^ and that 30.5% of MA beneficiaries have plans with narrow networks for primary care, 43.1% for psychiatry, and 83.2% for mental and behavioral health.^15^ In oncology, directory-based analyses suggest that 41% of plans exclude NCI-designated comprehensive cancer centers from their network.^16^

However, directory-based (ie, nominal) networks may misrepresent both network participation and usable access because organization and clinician directories are often inaccurate or outdated; even when organizations and clinicians are formally in network, beneficiaries may face barriers to realized access due to travel burden, appointment availability, and referral pathways. These limitations support a focus on effective networks based on realized care, which the Medicare Payment and Advisory Commission (MedPAC) has emphasized as a complementary and policy-relevant approach to assess beneficiary access beyond directory listings.^17^ While 1 prior study used Medicare Part D data to characterize effective primary care physician networks,^18^ no studies to our knowledge have evaluated effective oncology networks under MA. To address these gaps, this study leverages population-based cancer registry and Medicare claims data to evaluate trends in effective oncology organization and oncologist networks under MA plans from 2016 to 2019 and compare network breadth by metropolitan status and MA plan type.

## Methods

This cross-sectional study was deemed exempt by the Morehouse School of Medicine Institutional Review Board, which waived the informed consent requirement because this study used a secondary, limited dataset. The Strengthening the Reporting of Observational Studies in Epidemiology (STROBE) reporting guideline for cross-sectional studies was followed.

### Study Design and Sample

We used the Surveillance, Epidemiology, and End Results (SEER)–Medicare linkage, which includes Medicare beneficiaries newly diagnosed with cancer through 2019, with Medicare administrative data (fee-for-service claims and MA encounter data) from 2016 through 2019.^19^ SEER captures cancer characteristics, demographics, and neighborhood-level characteristics and covers approximately 35% of the US population.^20^ More than 95% of newly diagnosed patients with cancer aged 65 years or older were linked with Medicare data.^20^ Medicare administrative data include monthly Medicare enrollment and capture health care utilization incurred by beneficiaries. We used the Medicare Data on Provider Practice and Specialty^21^ to identify clinician specialty and affiliated organizations via tax identification numbers (TINs).

We extracted MA encounter records for all beneficiaries diagnosed with breast, lung, colorectal, prostate, pancreatic, bladder, blood (melanoma, leukemia, or lymphoma), or kidney cancer from 2010 to 2019, with 1 month of MA coverage or longer from 2016 through 2019 (n = 807 580). We included beneficiaries diagnosed from 2010 through2019 to capture both incident and prevalent cancer cases involving oncology use during the study period (2016-2019). Cancer sites were prespecified based on data use agreement, representing the most common malignant neoplasms (nearly 70% of all cancer cases) in the US. Because our objective was to characterize effective oncology networks at the plan level rather than to estimate patient-level outcomes, we included all observed oncology use by eligible beneficiaries regardless of survival time. Monthly MA enrollment information was used to determine, for each beneficiary-month, whether the beneficiary was enrolled in MA or TM. For MA-enrolled months, we used the contract identifier (ID) and plan benefit package (PBP) ID to assign beneficiaries to plans and then constructed network measures at the contract–PBP–county-year level.^22^ We restricted the analytic sample to plans with 3 or more beneficiaries with cancer in a given year to balance the need for stable estimation of plan-level oncology organization and oncologist networks and the need to retain smaller and rural plans. Sensitivity analyses using a higher threshold of at least 5 beneficiaries showed consistent results.

Notably, although organizational National Provider Identifiers (NPIs) and TINs were consistently populated in MA encounter files, individual NPIs exhibited nonnegligible and nonrandom missingness (eTable 1 in Supplement 1), with certain plans having 100% missing individual NPIs (eFigure 1 in Supplement 1). Therefore, our primary analysis was restricted to plans without complete NPI missingness across all encounter files.

Among included MA plans, we further identified special needs plans (SNPs) using a publicly available plan ID list.^23^ SNPs are specialized MA plans for beneficiaries with greater care coordination needs, such as those with dual eligibility for Medicaid or those with chronic conditions.^24,25^ Because SNPs differ from regular MA plans in both enrolled populations and plan structure, we analyzed SNPs separately from regular MA plans. Sample derivation is shown in eFigure 2 in Supplement 1.

### Effective Oncology Organization and Oncologist Network Metrics

The Centers for Medicare & Medicaid Services enforces MA network adequacy requirements for the number of organizations and clinicians as well as travel time and distance standards for 29 clinician specialty types and 14 facility specialty types,^26^ including 2 oncology-specific specialties: medical or surgical oncology and radiation oncology. In practice, however, MA contract negotiation often occurs at the organization level rather than the individual clinician level. Therefore, we evaluated plan-county-year network metrics on both levels. Oncology organizations are defined as those with TINs in the Medicare Data on Provider Practice and Specialty file with which medical, surgical, or radiation oncologists reported affiliations.

We operationalized MA effective networks using realized use in MA encounter data, consistent with MedPAC’s workplan definition of effective networks based on the network “used by MA enrollees.” Specifically, within each contract–PBP–county-year, we defined an oncology organization or oncologist to be part of a plan’s effective network if they had more than 1 oncology visit recorded in MA encounter data for beneficiaries enrolled in that plan during the year. This use-defined measurement reflects organizations or oncologists actually accessed under MA as the payer of record.

We defined the oncology organization and oncologist universe using the set of organizations and oncologists found in fee-for-service claims from 2016 through 2019 among TM beneficiaries residing in the same county-year to approximate the local oncology organization and oncologist opportunity set under a non–network-restricted coverage model.

Effective oncology network breadth was defined as the share of oncology organizations or oncologists accessed by MA beneficiaries relative to those accessed by TM beneficiaries in the same county-year. For each contract–PBP–county-year, we measured (1) the number of oncology organizations billing more than 1 visit to an MA plan divided by the number of oncology organizations accessed by TM beneficiaries and (2) the number of medical or surgical oncologists and radiation oncologists billing more than 1 visit to an MA plan divided by the corresponding counts among TM beneficiaries. We considered MA plans to have narrow networks if their effective network breadth was 25% or less of the breadth of oncology organizations or oncologists serving TM beneficiaries in the county.^15^ Lastly, we evaluated effective access to NCI-designated comprehensive cancer centers, defined as more than 1 visit recorded under an MA plan at any NCI-designated center. Construction of each network measure is detailed in eTable 2 in Supplement 1.

We included county metropolitan status based on 2013 Rural-Urban Continuum Codes.^27^ SEER racial and ethnic categories included Hispanic, non-Hispanic Black, non-Hispanic White, non-Hispanic other (American Indian or Alaska Native, Asian or Pacific Islander, or other unspecified), and unknown. This information is provided to describe the sample characteristics of the cohort contributing to the network breadth metrics. MA plans were classified as health maintenance organization (HMO), local preferred provider organization (PPO), regional PPO, point-of-service (POS) plan, or other (medical savings account, private fee-for-service plan, Section 1876 Cost Plan, or Program of All-Inclusive Care for the Elderly).

### Statistical Analysis

We assessed effective oncology network measures separately for regular MA plans and SNPs. Annual trends in each network metric were plotted for 2016 through 2019. We also compared network metrics by metropolitan status and MA plan type using univariate linear regression for continuous metrics of network breadth and logistic regression for binary metrics of effective access to NCI-designated centers. Finally, we mapped the county-level percentage of MA plans with effective access to an NCI-designated center.

To ensure robustness of our analysis, we conducted sensitivity analyses (1) restricted to plans missing 10% or fewer hospital stays and less than ±10% difference in ambulatory and ED visits using previously published algorithms^28,29^; (2) restricted to plans with 5 or more beneficiaries diagnosed with cancer in a given year; (3) stratifying plans by more than 100 and 100 or fewer beneficiaries identified with cancer under the MA plans; and (4) excluding plans in California, given the high penetration of Kaiser Permanente plans.

Data analyses were performed in SAS version 9.4 (SAS Institute Inc) and RStudio version 4.4.1 (R Project for Statistical Computing). Statistical significance was evaluated at a *P* < .05 threshold using 2-sided tests. Data were analyzed between March 1, 2025, and April 1, 2026.

## Results

A total of 807 580 MA beneficiaries (mean [SD] age, 70.4 [9.0] years; 52.1% [n = 420 662] male) were identified for the network analysis, representing 23 255 (from 980 counties) plan-year observations for regular MA plans and 17 716 (from 901 counties) for SNPs from 2016 through 2019. The overall racial and ethnic composition of MA beneficiaries included 14.5% (n = 116 830) Hispanic, 14.6% (n = 117 711) non-Hispanic Black, 64.5% (n = 520 845) non-Hispanic White, and 5.5% (n = 44 359) non-Hispanic other (other category included American Indian or Alaska Native, Asian or Pacific Islander, or other unspecified) individuals; race and ethnicity were unknown for 1.0% (n = 7835). The median (IQR) number of beneficiaries with cancer across MA plans was 16 (<11 to 108). We included 1 269 395 TM beneficiaries (mean [SD] age, 70.8 [9.3] years; 681 955 males [53.7%]; 108 145 Hispanic [8.5%], 125 864 non-Hispanic Black [9.9%], 967 034 non-Hispanic White [76.2%], and 56 157 non-Hispanic other [4.4%] individuals; race and ethnicity were unknown for 12 195 individuals [1.0%]) to construct a TM utilization-based oncology organization and oncologist universe. The number of regular MA plans increased from 5339 to 6053 during the study period. There was a larger increase in SNPs, from 3206 in 2016 to 6638 in 2019. Among 23255 regular MA plans, 25.7% (n = 5974) were HMOs, 47.9% (n = 11 142) were local PPOs, 17.1% (n = 3979) were regional PPOs, and 4.3% POS (n = 1006) plans; among 17716 SNPs, 48.2% (n = 8540) were HMOs, followed by 21.1% (n = 3731) local PPOs, 24.4% (n = 4329) regional PPOs, and 4.9% (n = 875) POS plans. Most regular MA plans (68.7% [n = 15 972 of 23 255]) and SNPs (69.3% [n = 12 277 of 17 716]) were from metropolitan counties (Table; eTable 3 in Supplement 1).

**Table.** MA Plan Statistics and Average Effective Oncology Organization and Oncologist Network Metrics, 2016 through 2019

Statistic or metric	Plan-year observations, No. (%)		
	Total (N = 40 971)	Regular MA plan (n = 23 255)	SNP (n = 17 716)
Beneficiaries with cancer identified under the plan, median (IQR)	16 (<11 to 108)	16 (<11 to 112)	16 (<11 to 105)
Year			
2016	8545 (20.9)	5339 (23.0)	3206 (18.1)
2017	9227 (22.5)	5643 (24.3)	3584 (20.2)
2018	10 508 (25.6)	6220 (26.7)	4288 (24.2)
2019	12 691 (31.0)	6053 (26.0)	6638 (37.5)
Medicare plan type			
HMO	14 514 (35.4)	5974 (25.7)	8540 (48.2)
Local PPO	14 873 (36.3)	11 142 (47.9)	3731 (21.1)
Regional PPO	8308 (20.3)	3979 (17.1)	4329 (24.4)
POS plan	1881 (4.6)	1006 (4.3)	875 (4.9)
Other^a^	1395 (3.4)	1154 (5.0)	241 (1.4)
Metropolitan status			
Metropolitan	28 249 (68.9)	15 972 (68.7)	12 277 (69.3)
Nonmetropolitan	12 722 (31.1)	7283 (31.3)	5439 (30.7)
Effective network breadth for oncology organizations, mean (SD), %	12.2 (12.7)	12.0 (12.7)	12.4 (12.6)
Plans with narrow network for oncology organizations, mean (SD), %	86.1 (34.6)	86.7 (33.9)	85.1 (35.6)
Effective network breadth for medical or surgical oncologists, mean (SD), %	7.0 (9.2)	6.8 (9.6)	7.2 (8.6)
Plans with narrow network for medical or surgical oncologists, mean (SD), %	95.6 (20.5)	96.0 (19.6)	95.1 (21.6)
Effective network breadth for radiation oncologists, mean (SD), %	12.1 (14.6)	11.6 (13.8)	12.7 (15.5)
Plans with narrow network for radiation oncologists, mean (SD), %	88.0 (32.5)	89.0 (31.3)	86.6 (34.1)
Plans with effective access to NCI-designated cancer center	9607 (23.4)	5983 (25.7)	3624 (20.5)

Abbreviations: HMO, Health Maintenance Organization; MA, Medicare Advantage; POS, point of service; PPO, preferred provider organization; SNP, special needs plan.

^a^ Other includes medical savings account, private fee-for-service plan, Section 1876 Cost Plan, or Program of All-Inclusive Care for the Elderly.

### Overall Effective Oncology Organization and Oncologist Network

**Figure 1.**
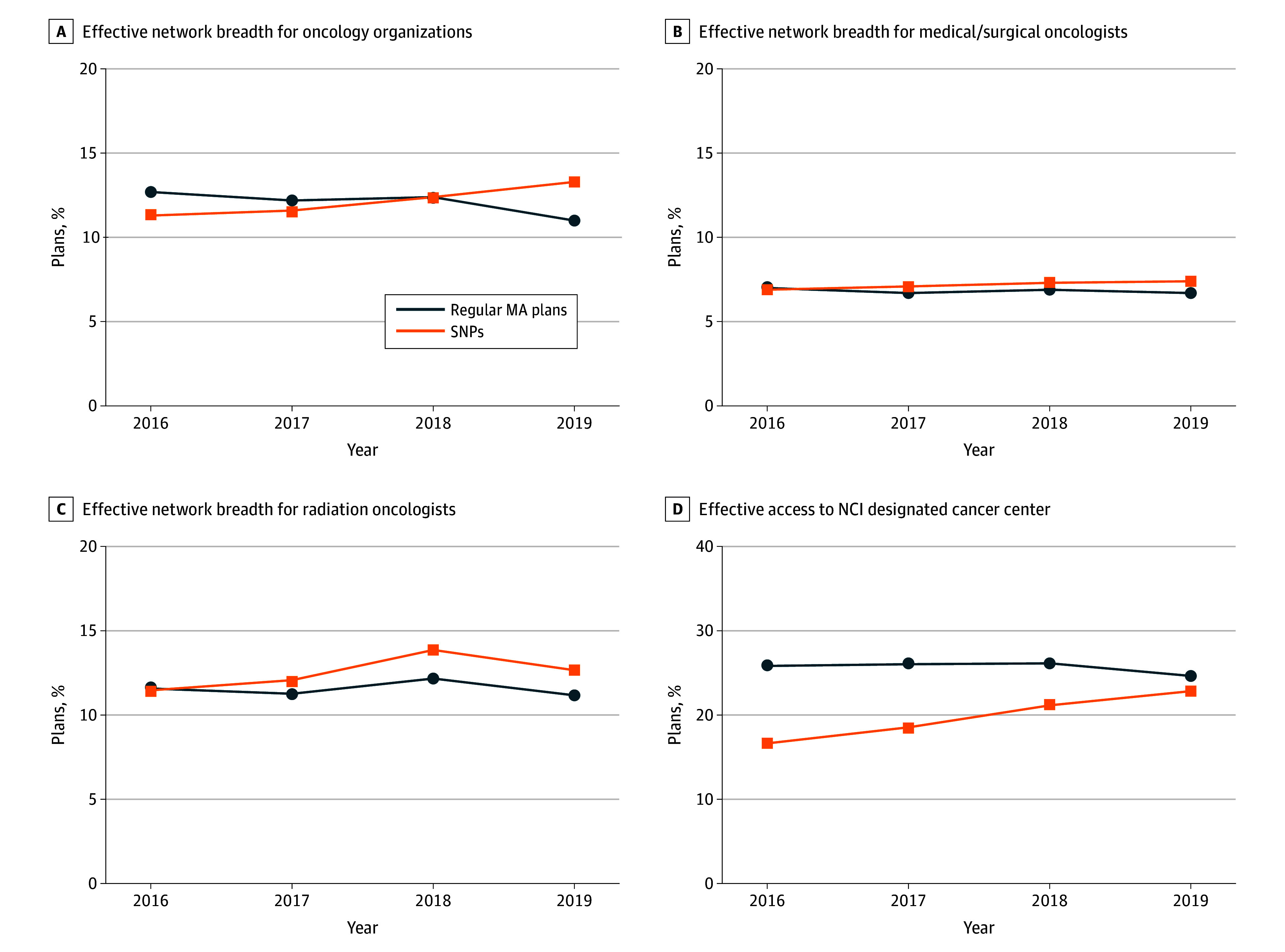
Line Graphs of Trends of Effective Oncology Organization and Oncologist Network Metrics MA indicates Medicare Advantage; NCI, National Cancer Institute; SNP, special needs plan. ^a^*P* < .05 compared to with reference year (2016).

Across counties, the mean number of oncology organizations with 1 or more visits by TM beneficiaries ranged between 98 and 120 compared with fewer than 11 (exact number suppressed per data use agreement) accessed by MA beneficiaries from 2016 through 2019 (eTable 4 in Supplement 1). Mean (SD) effective oncology network for oncology organizations was 12.0% (12.7%) for MA plans and 12.4% (12.6%) for SNPs (Table). The mean (SD) percentages remained stable during the study period: 12.7% (12.8%) in 2016 and 11.0% (11.7%) in 2019 for regular MA plans, and 11.3% (11.3%) in 2016 and 13.3% (13.1%) in 2019 for SNPs (Figure 1). More than a mean (SD) 86.7% (33.9%) of regular MA plans were classified as having a narrow effective network, ranging from 86.0% (34.8%) to 88.9% (31.4%) in 2016 through 2019; 85.1% (35.6%) of SNPs had narrow networks, ranging from 87.6% (32.9%) to 82.9% (37.7%) in 2016 through 2019 (eFigure 3 in Supplement 1).

The mean number of medical or surgical oncologists with more than 1 visit by TM beneficiaries ranged between 168 and 215, relative to a mean of fewer than 11 accessed under MA in 2016 through 2019. For radiation oncologists, the corresponding counts were 48 to 60 under TM and fewer than 11 under MA (eTable 4 in Supplement 1). Mean (SD) effective network breadth was 6.8% (9.6%) under regular MA plans and 7.2% (8.6%) under SNPs for medical or surgical oncologists and 11.6% (13.8%) under regular MA plans and 12.7% (15.5%) under SNPs for radiation oncologists. Similarly high percentages of plans had narrow networks for medical or surgical oncologists (mean [SD] 96.0% [19.6%] among regular MA plans and 95.1% [21.6%] among SNPs) and radiation oncologists (mean [SD] 89.0% [31.3%] among regular MA plans and 86.6% [34.1%] among SNPs) (Table and Figure 1; eFigure 3 in Supplement 1).

Across regular MA plans, 25.7% (n = 5983 of 23 255) included NCI-designated centers, which remained relatively stable during the study period. Across SNPs, 20.5% (n = 3624 of 17 716) included NCI-designated centers, increasing from 16.7% to 22.9% from 2016 through 2019 (Figure 1).

### Network Comparison by Metropolitan Status and MA Plan Type

**Figure 2.**
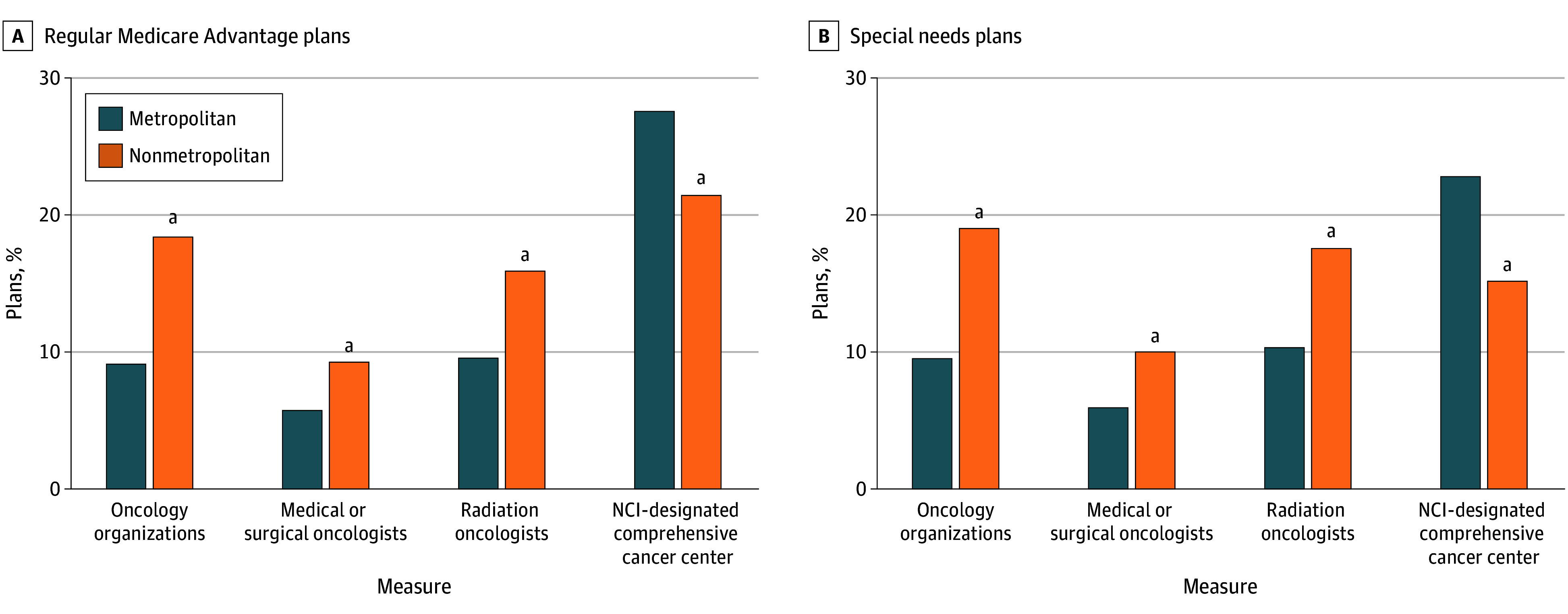
Bar Graphs of Effective Oncology Organization and Oncologist Network Comparison by Metropolitan Status NCI indicates National Cancer Institute. ^a^*P* < .05 compared with metropolitan counties.

The mean number of oncology organizations visited by TM beneficiaries was smaller in nonmetropolitan (range, 27-47) than in metropolitan (range, 128-156) countiesfrom 2016 through 2019. Under MA plans during the same time frame, the number of oncology organizations visited by beneficiaries in nonmetropolitan and metropolitan counties was similar (all fewer than 11) (eTable 4 in Supplement 1) in 2016-2019. Accordingly, effective MA network breadth for oncology organizations was smaller in metropolitan than in nonmetropolitan counties for both regular MA plans (9.1% vs 18.5%) and SNPs (9.5% vs 19.0%; *P* < .001). We also observed smaller effective networks in metropolitan than in nonmetropolitan counties for medical or surgical oncologists and radiation oncologists. However, the percentage of MA plans including NCI-designated centers was higher among metropolitan than among nonmetropolitan counties for both regular MA plans (27.7% vs 21.5%) and SNPs (22.8% vs 15.2%; *P* < .001) (Figure 2).

**Figure 3.**
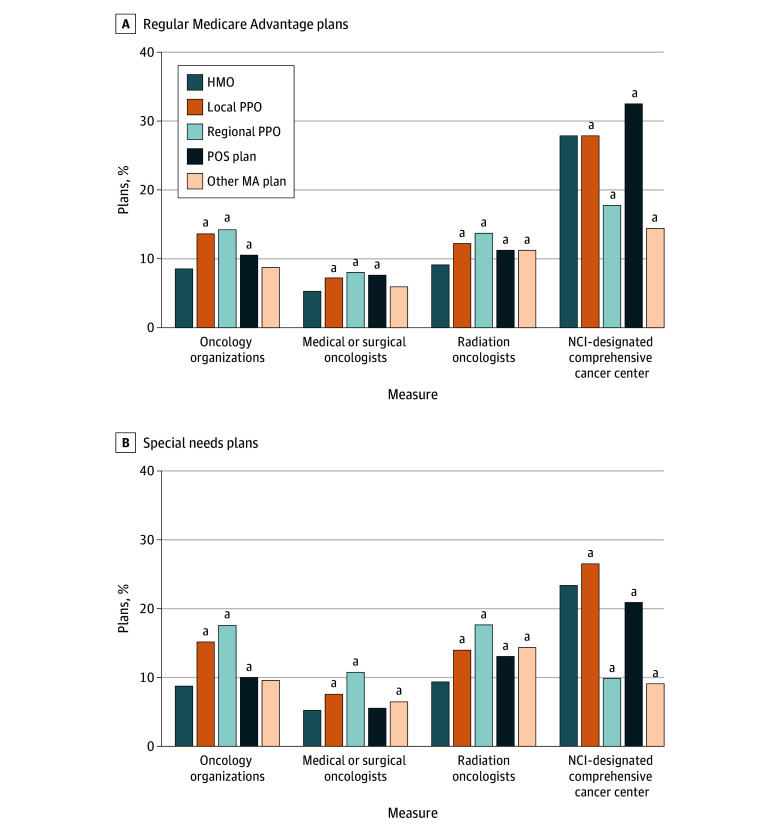
Bar Graphs of Effective Oncology Organization and Oncologist Network Comparison by Medicare Advantage (MA) Type HMO indicates health maintenance organization; NCI, National Cancer Institute; POS, point of service; PPO, preferred provider organization. ^a^*P* value <.05 compared with HMO plans.

Comparison across MA plan types showed generally narrower networks for HMOs than other plan types. Among regular MA plans, effective network breadth for oncology organizations was 8.5% for HMOs, 13.6% for local PPOs, 14.2% for regional PPOs, and 10.5% for POS plans (*P* < .001 vs HMOs). Effective access to NCI-designated centers was 27.9% among HMOs and significantly higher for POS plans (32.5%; *P* < .001) but lower for regional PPOs (17.7%; *P* < .001). Similarly, lower access to NCI-designated centers was observed for regional PPOs compared with HMOs (Figure 3).

### Geographic Variation in Inclusion of NCI-Designated Comprehensive Cancer Centers

**Figure 4.**
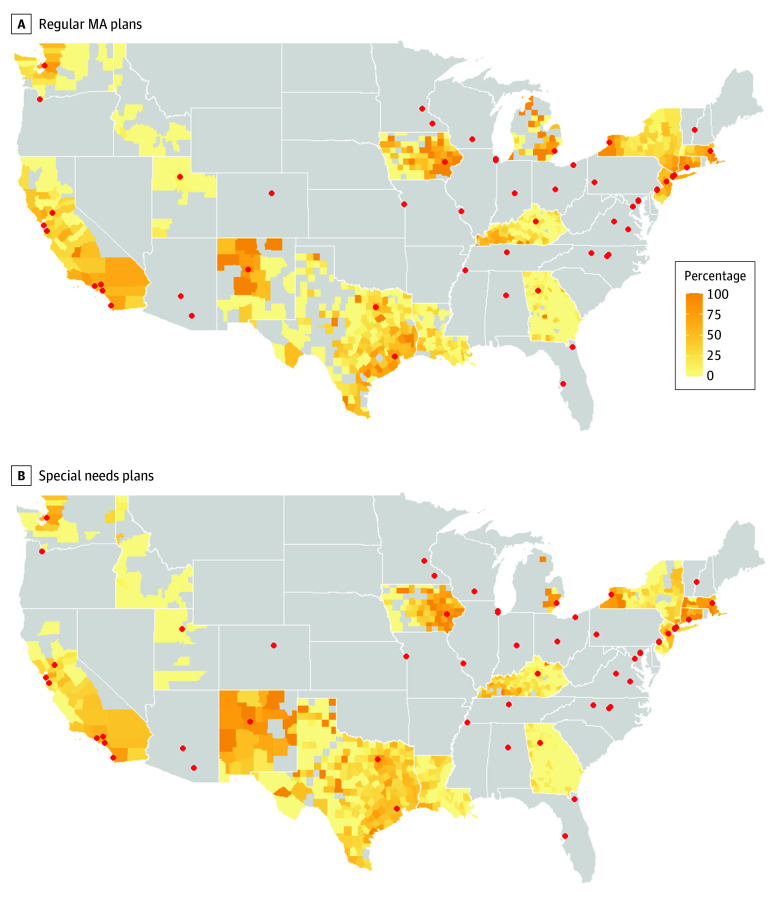
Maps of County-Level Percentage of Medicare Advantage (MA) Plans Covering National Cancer Institute (NCI)–Designated Comprehensive Cancer Centers in 2019 Dots represent NCI-designated comprehensive cancer centers.

Considerable variation existed in the percentages of MA plans including NCI-designated centers across counties, ranging from 0% to 100%. Certain regions, such as New York State and southern California, showed higher percentages of MA plans including NCI-designated centers. However, many counties in Georgia and Utah showed relatively low percentages of MA plans including NCI-designated centers (Figure 4). For example, among the 154 counties our analysis covered in Georgia, 115 (74.7%) had 0% MA plans with recorded visits to an NCI-designated cancer center.

### Sensitivity Analysis

Sensitivity analyses restricted to plans with fewer than 10% missing hospital stays and less than ±10% difference in ambulatory and ED visits were comparable to the primary sample. For example, the mean (SD) share of oncology organizations accessed under MA was 12.2% (12.9%) for regular MA plans and 13.9% (13.1%) for SNPs (compared with 12.0% [12.7%] for regular MA plans and 12.4% [12.6%] for SNPs in the primary analysis) (eFigure 4 in Supplement 1). Sensitivity analyses restricted to plans with 5 or more beneficiaries with cancer produced similar results; the mean (SD) share of oncology organizations accessed under MA was 12.7% (13.0%) for regular MA plans and 13.1% (12.8%) for SNPs (eFigure 5 in Supplement 1). When stratified by cancer volume (with 100 or more vs fewer than 100 beneficiaries with cancer), most network measures were similar, except that NCI-designated center coverage was higher in the stratum of 100 or more (eFigure 6 in Supplement 1). Excluding plans in California also produced similar results to the primary analysis (eFigure 7 in Supplement 1).

## Discussion

To our knowledge, our study provides the first population-based assessment of effective oncology networks under MA. Within SEER regions, MA beneficiaries accessed only 12.0% of oncology organizations and 6.8% to 11.6% of medical or surgical and radiation oncologists who served TM beneficiaries, with little change from 2016 through 2019. Accordingly, approximately 90% of MA plans had narrow networks, and fewer than 30% covered services at an NCI-designated centers.

These findings align with and extend existing research documenting narrow nominal MA networks.^12,14,15^ For example, 1 directory-based analysis reported that 83.2% of MA beneficiaries had plans with narrow networks for mental and behavioral health clinicians.^15^ Although oncology has not been previously examined, we found that approximately 90% of MA plans had narrow effective oncology networks. Effective networks reflect the combined influence of (1) MA plan network design, including selective contracting, use management, and gatekeeping, and (2) noninsurance barriers that affect realized care even when organizations or clinicians are nominally available, including geographic proximity, appointment availability, referral pathways, and patient preferences. The relatively limited and geographically concentrated oncology workforce may further compress network breadth in some markets.^30^ Notably, our estimates of effective access to NCI-designated centers were lower than prior directory-based estimates,^14^ suggesting that directory-based nominal networks may overstate usable access and reinforce the importance of utilization-based network measures.

We also extend limited evidence on regular MA plans vs SNPs, which shows generally comparable network breadth, with slightly lower NCI-designated center coverage among SNPs in earlier years. This similarity may reflect the already highly constrained nature of oncology networks under MA.

Despite substantial increases in MA enrollment,^4^ oncology network breadth remained largely stable over time, which suggests structural features of MA contracting may contribute to network constraints. Contracting between organizations and/or clinicians and plans is typically conducted through multiyear agreements, which supports continuity of care and ensures operational efficiency but could limit opportunities to expand oncology networks and constrain patients’ ability to seek care from NCI-designated centers and/or innovative cancer treatments. These constraints are particularly consequential because beneficiaries often enroll in MA without anticipating future cancer care needs and only encounter network limitations after a cancer diagnosis. At that point, switching back to TM can be difficult due to medical underwriting rules for Medigap coverage,^31^ and out-of-network access to oncology organizations or oncologists may carry prohibitive costs during an already medically vulnerable time. Recent reports of academic hospitals and NCI-designated centers terminating MA contracts further highlight concerns about access erosion^32,33,34,35^ and encourage future research on how these contract disruptions affect oncology care delivery and outcomes.

We observed meaningful variation by geography and plan type. Although metropolitan counties had narrower proportional networks, this likely reflects larger denominators of the oncology workforce (average of 134 oncology organizations in metropolitan vs 39 in nonmetropolitan counties). However, plans in nonmetropolitan counties had significantly lower NCI coverage despite broader proportional networks, potentially reflecting the geographic clustering of NCI-designated centers in metropolitan areas.^36^ Differences by MA plan type are clinically and policy relevant because of distinct benefit designs and care management structures that shape organization and clinician contracting and patient referrals. HMOs typically operate with more restrictive networks and stronger gatekeeping, which can facilitate care coordination and price negotiation but may also limit access to subspecialists and NCI-designated centers^37^; PPOs and POS plans showed broader networks, aligned with their generally greater flexibility for out-of-network care, although they were still limited in absolute terms.

These findings have important policy implications. The Centers for Medicare and Medicaid Services’ network adequacy standards requiring only 1 physician per specialty within a service area are unlikely to ensure meaningful access for complex conditions such as cancer.^26^ While restricting organization or clinician networks may promote efficiency or concentrate care among higher-quality organizations or oncologists,^37^ empirical evidence is needed to inform policy standards, whether based on network breadth, access to NCI-designated centers, or other access metrics, to ensure equitable outcomes for MA beneficiaries. Our results also reinforce MedPAC’s call for greater transparency and monitoring of effective organization or oncologist access.^17^ Narrow effective oncology networks could limit access to subspecialists, second opinions, novel cancer therapies, and clinical trials, particularly for rural beneficiaries or HMO enrollees. Because effective networks reflect both strategic contracting and access barriers, the welfare implications of narrower effective networks remain uncertain. Understanding how both nominal and effective oncology network breadth influences timely receipt of guideline-concordant treatment, survival, and patient-reported outcomes is therefore an important priority for future research.

### Limitations

Our study has limitations. First, we focused on 8 common cancers, representing approximately 70% of incident cancer cases, which may not capture all beneficiaries receiving oncology care. Future studies using broader Medicare data are needed to validate and extend our findings. Second, our networks reflect organizations or oncologists accessed under MA as the payer of record. For many enrollees, including SNP enrollees, Medicare represents the primary payer for oncology services. For enrollees who obtain oncology care through other payers (eg, VA or private insurance), undercapture of oncology organizations or oncologists under MA is limited and would occur primarily when an organization or clinician is accessed exclusively through non-MA payers for all relevant enrollees in a plan–county-year. Conversely, our realized care approach may capture some out-of-network care. Our network inclusion requiring at least 2 visits per organization or oncologist reduces but does not eliminate this possibility. Third, because MA enrollment is not random, the composition of beneficiaries with cancer observed in MA vs TM may differ in disease severity and comorbidity burden, which may influence realized care. Although this population is expected to have substantial cancer care needs, our analyses describe MA-realized networks among enrolled patients with cancer and do not address whether MA network characteristics change enrollment. Fourth, we did not evaluate clinical outcomes; our findings describe differences in effective networks but do not establish whether narrower networks affect treatment patterns or cancer outcomes. Finally, our estimations were based on counties within SEER regions from 2016 through 2019, when MA penetration was lower than in recent years, and may not be fully generalizable to current enrollment levels or to non-SEER regions with different oncology workforce distributions or MA penetration patterns.

## Conclusions

In this cross-sectional study of MA beneficiaries with cancer, we found that effective oncology organization and oncologist networks were narrow and largely unchanged during the study period, with approximately one-quarter of plans including an NCI-designated comprehensive cancer center. Geographic and plan type differences further highlight substantial inequities in oncology care access across the MA program. Findings underscore the need for improved transparency, regulatory monitoring, and evaluation of how MA oncology organization and oncologist networks impact cancer care delivery, access to innovative treatment, and patient outcomes.

## Notes

Supplement 1. **eTable 1.** Percent of Claims With Missing Individual NPI by File Type **eTable 2.** Study Variable Constructions **eTable 3.** Medicare Beneficiaries’ Characteristics Included in Network Analysis **eTable 4.** Number of Oncology Organizations and Oncologists Seen by Traditional Medicare and Medicare Advantage Beneficiaries **eFigure 1.** Missingness of Provider Identifiers on Medicare Advantage Plan Level **eFigure 2.** Sample Flow Chart **eFigure 3.** Trend of Narrow Effective Oncology Provider Network **eFigure 4.** Trend of Effective Oncology Provider Network Metrics Among Plans With <10% Missing Inpatient and/or Outpatient Visits **eFigure 5.** Trend of Effective Oncology Provider Network Metrics Among Plans With At Least 5 Beneficiaries With Cancer **eFigure 6.** Trend of Effective Oncology Provider Network Metrics Stratifying by >100 and ≤100 Beneficiaries With Cancers Under the Medicare Advantage Plans **eFigure 7.** Trend of Effective Oncology Provider Network Metrics Excluding Plans in California

Supplement 2.
Data Sharing Statement
