# Supplementary material for: Oncology Organization and Oncologist Networks Under Medicare Advantage Plans
Source: JAMA Netw Open. 2026 Jun 15;9(6):e2618507. doi: 10.1001/jamanetworkopen.2026.18507 (PMC13270271; doi:10.1001/jamanetworkopen.2026.18507)
Supplement: Supplement 1. — eTable 1. Percent of Claims With Missing Individual NPI by File Type eTable 2. Study Variable Constructions eTable 3. Medicare Beneficiaries’ Characteristics Included in Network Analysis eTable 4. Number of Oncology Organizations and Oncologists Seen by Traditional Medicare and Medicare Advantage Beneficiaries eFigure 1. Missingness of Provider Identifiers on Medicare Advantage Plan Level eFigure 2. Sample Flow Chart eFigure 3. Trend of Narrow Effective Oncology Provider Network eFigure 4. Trend of Effective Oncology Provider Network Metrics Among Plans With <10% Missing Inpatient and/or Outpatient Visits eFigure 5. Trend of Effective Oncology Provider Network Metrics Among Plans With At Least 5 Beneficiaries With Cancer eFigure 6. Trend of Effective Oncology Provider Network Metrics Stratifying by >100 and ≤100 Beneficiaries With Cancers Under the Medicare Advantage Plans eFigure 7. Trend of Effective Oncology Provider Network Metrics Excluding Plans in California [file jamanetwopen-e2618507-s001.pdf]

Supplemental Online Content

Hu X, Kwon Y, Fan Q, et al. Oncology organization and oncologist networks under Medicare Advantage plans. *JAMA Netw Open*. 2026;9(6):e2618507.
doi:10.1001/jamanetworkopen.2026.18507

eTable 1. Percent of Claims With Missing Individual NPI by File Type

eTable 2. Study Variable Constructions

eTable 3. Medicare Beneficiaries' Characteristics Included in Network Analysis

eTable 4. Number of Oncology Organizations and Oncologists Seen by Traditional Medicare and Medicare Advantage Beneficiaries

eFigure 1. Missingness of Provider Identifiers on Medicare Advantage Plan Level

eFigure 2. Sample Flow Chart

eFigure 3. Trend of Narrow Effective Oncology Provider Network

eFigure 4. Trend of Effective Oncology Provider Network Metrics Among Plans With <10% Missing Inpatient and/or Outpatient Visits

eFigure 5. Trend of Effective Oncology Provider Network Metrics Among Plans With At Least 5 Beneficiaries With Cancer

eFigure 6. Trend of Effective Oncology Provider Network Metrics Stratifying by >100 and ≤100 Beneficiaries With Cancers Under the Medicare Advantage Plans

eFigure 7. Trend of Effective Oncology Provider Network Metrics Excluding Plans in California

This supplemental material has been provided by the authors to give readers additional information about their work.

© 2026 Hu X, et al. *JAMA Network Open*.

eTable 1. Percent of Claims with Missing Individual NPI by File Type

File Type	Year	Total Number of Claims	% Missing Individual NPI[1]
Inpatient Base	2016	360096	27.3%
Inpatient Base	2017	443585	29.9%
Inpatient Base	2018	493123	21.1%
Inpatient Base	2019	577865	21.3%
Inpatient Base	2020	532413	23.4%
Inpatient Base	2021	611333	23.4%
Carrier Base	2016	30245316	36.5%
Carrier Base	2017	34603832	32.9%
Carrier Base	2018	38681563	32.8%
Carrier Base	2019	43803264	32.8%
Carrier Base	2020	42588508	33.3%
Carrier Base	2021	49917452	31.1%
Outpatient Base	2016	4279016	14.5%
Outpatient Base	2017	4800340	14.3%
Outpatient Base	2018	5388635	14.1%
Outpatient Base	2019	6113449	12.4%
Outpatient Base	2020	5902150	11.5%
Outpatient Base	2021	7017625	10.5%
HHA Base	2016	325623	16.4%
HHA Base	2017	374928	15.1%
HHA Base	2018	438859	20.8%
HHA Base	2019	562823	21.1%
HHA Base	2020	662299	17.6%
HHA Base	2021	750216	16.9%
SNF Base	2016	78310	27.7%
SNF Base	2017	90247	26.5%
SNF Base	2018	98301	24.6%
SNF Base	2019	101579	15.2%
SNF Base	2020	85638	15.3%
SNF Base	2021	99425	13.8%

Notes: NPI=National Provider Identifier; HHA=Home Health Agency; SNF=Skilled Nursing Facility.

[1] We used the attending physician NPIs for inpatient, outpatient, HHA and SNF files, and rendering physician NPIs for carrier files.

© 2026 Hu X, et al. *JAMA Network Open*.

eTable 2. Study Variable Constructions

Variable	Definition
Total TINs with oncologists accessed under MA	Total number of TINs with oncologists (with special codes “82”, “83”, “90”, “91”, “92”, “98” identified from MD-PPAS files) accessed by MA beneficiaries within a specific county. TINs had to appear on >1 MA recorded visits under a specific MA plan.
Total TINs with oncologists within a county	Total number of TINs with oncologists (with special codes “82”, “83”, “90”, “91”, “92”, “98” identified from MD-PPAS files) accessed by TM beneficiaries within a specific county. TINs had to appear on >1 FFS recorded visits.
Effective network breadth for oncology organizations	Total TINs with oncologists with >1 MA recorded visits under the MA plan/ Total TINs with oncologists with >1 FFS recorded visits within a county
Percentage of plans with narrow network for oncology organizations	Effective network breadth for oncology organizations < 25%
Effective access to NCI designated cancer center	Medicare provider ID is flagged as an NCI designated cancer center from the hospital characteristic file with >1 MA recorded visits under a specific MA plan.
Total number of Medical/Surgical Oncologist accessed under MA	Total number of Medical/Surgical/Hematologic/Gynecologic oncologists (with special codes of “82”, “83”, “90”, “91”, “98” identified from MD-PPAS files) accessed by MA beneficiaries within a specific county. Oncologists had to appear on >1 MA recorded visits under a specific MA plan.
Total number of Medical/Surgical Oncologist within a county	Total number of Medical/Surgical/Hematologic/Gynecologic oncologists (with special codes “82”, “83”, “90”, “91”, “98” identified from MD-PPAS files) accessed by TM beneficiaries within a specific county. Oncologists had to appear on >1 FFS recorded visits.
Effective network breadth for Medical/Surgical Oncologists	Total Medical/Surgical/Hematologic/Gynecologic oncologists with >1 MA recorded visits under the MA plan / Total Medical/Surgical/Hematologic/Gynecologic oncologists with >1 FFS recorded visits within a county
Percentage of plans with narrow network for Medical/Surgical Oncologists	Effective network breadth for Medical/Surgical Oncologists < 25%
Total number of Radiation Oncologist accessed under MA	Total number of radiation oncologists (with special codes of “92” identified from MD-PPAS files) accessed by MA beneficiaries within a specific county. Oncologists had to appear on >1 MA recorded visits under a specific MA plan.
Total number of Radiation Oncologist within a county	Total number of radiation oncologists (with special codes of “92” identified from MD-PPAS files) accessed by TM beneficiaries within a specific county. Oncologists had to appear on >1 FFS recorded visits.
Effective network breadth for Radiation Oncologists	Total Radiation oncologists with >1 MA recorded visits under the MA plan / Total Radiation oncologists with >1 FFS recorded visits within a county
Percentage of plans with narrow network for Radiation Oncologists	Effective network breadth for Radiation Oncologists < 25%

Notes: MA = Medicare Advantage; TM = Traditional Medicare.

© 2026 Hu X, et al. *JAMA Network Open*.

eTable 3. Medicare Beneficiaries' Characteristics Included in Network Analysis

	Traditional Medicare	Medicare Advantage
Total N, N (Row %)	1269395 (61.1)	807580 (38.9)
Age at diagnosis, Mean (SD)	70.82 (9.3)	70.38 (9.0)
Sex, N (Col %)		
Male	681955 (53.7)	420662 (52.1)
Female	587440 (46.3)	386918 (47.9)
Cancer site, N (Col %)		
Bladder	87568 (6.9)	52097 (6.5)
Breast	294587 (23.2)	203505 (25.2)
Colorectal	145766 (11.5)	95022 (11.8)
Hematologic	154966 (12.2)	90314 (11.2)
Kidney	62808 (5.0)	39768 (4.9)
Lung	173352 (13.7)	101986 (12.6)
Pancreas	38106 (3.0)	21899 (2.7)
Prostate	312242 (24.6)	202989 (25.1)
Race and ethnicity, N (Col %)		
Hispanic	108145 (8.5)	116830 (14.5)
Non-Hispanic White	967034 (76.2)	520845 (64.5)
Non-Hispanic Black	125864 (9.9)	117711 (14.6)
Non-Hispanic Other	56157 (4.4)	44359 (5.5)
Unknown	12195 (1.0)	7835 (1.0)
Marital Status, N (Col %)		
Married	420415 (33.1)	263551 (32.6)
Not married	279980 (22.1)	180884 (22.4)
Unknown	569000 (44.8)	363145 (45.0)
Metropolitan Status [a], N (Col %)		
Non-metropolitan	177164 (14.0)	71166 (8.8)
Metropolitan	1092128 (86.0)	736356 (91.2)
Unknown	103 (0.0)	58 (0.0)
Yost Index Quintiles [b], N (Col %)		
Q1	189162 (14.9)	151413 (18.8)
Q2	204299 (16.1)	135993 (16.8)
Q3	228974 (18.0)	151482 (18.8)
Q4	272382 (21.5)	174842 (21.7)
Q5	366168 (28.9)	187671 (23.2)
Unknown	8410 (0.7)	6179 (0.8)
Diagnosis Year, N (Col %)		
2010	101643 (8.0)	63373 (7.9)
2011	107950 (8.5)	66686 (8.3)
2012	106495 (8.4)	66690 (8.3)
2013	111332 (8.8)	69227 (8.6)
2014	115857 (9.1)	72424 (9.0)
2015	131642 (10.4)	84234 (10.4)
2016	153645 (12.1)	98941 (12.3)
2017	152172 (12.0)	98544 (12.2)
2018	147300 (11.6)	94892 (11.8)
2019	141359 (11.1)	92569 (11.5)

[a] Based on 2013 Rural Urban Continuum Code. Codes 1-3 were classified as metro, and Codes 4-9 were classified as non-metro.

[b] Yost index is a composite SES scores for census tracts based on Median household income, Median house value, Median rent, Percent below 150% of poverty line, Education Index, Percent working class, and Percent unemployed. Lower quintiles represent the lower SES groups. Details can be found: https://seer.cancer.gov/seerstat/databases/census-tract/index.html

© 2026 Hu X, et al. *JAMA Network Open*.

eTable 4. Number of Beneficiaries with Cancer under Medicare Advantage Plan and Number of Oncology Providers Seen by Medicare Advantage and Traditional Medicare Beneficiaries

	Regular MA plans				SNPs			
	**2016**	**2017**	**2018**	**2019**	**2016**	**2017**	**2018**	**2019**
Total number of beneficiaries with cancer under the MA plan								
Mean (SD)	491 (4300)	532 (3541)	734 (8252)	813 (6816)	775 (7320)	1059 (10254)	1038 (8079)	1041 (10651)
Median (IQR)	16 [<11, 99]	16 [<11, 111]	16 [<11, 120]	16 [<11, 126]	15 [<11, 90]	16 [<11, 100]	18 [<11, 114]	16 [<11, 108]
Total number of oncology organizations with >1 visit by MA beneficiaries								
Mean (SD) [1]	----	----	----	----	----	----	----	----
Median (IQR) [1]	----	----	----	----	----	----	----	----
Total number of oncology organizations with >1 visit by TM beneficiaries								
Mean (SD)	98 (106)	101 (105)	105 (107)	113 (109)	117 (138)	114 (133)	120 (140)	106 (125)
Median (IQR)	55 [29, 129]	58 [32, 136]	60 [33, 145]	73 [36, 164]	51 [26, 157]	50 [27, 148]	52 [29, 159]	48 [28, 139]
Total number of Medical/Surgical/Hematologic/Gynecologic Oncologist with >1 visit by MA beneficiaries								
Mean (SD) [1]	----	----	----	----	----	----	----	----
Median (IQR) [1]	----	----	----	----	----	----	----	----
Total number of Medical/Surgical/Hematologic/Gynecologic Oncologist with >1 visit by TM beneficiaries								
Mean (SD)	168 (183)	175 (190)	180 (198)	197 (207)	207 (242)	202 (241)	215 (259)	190 (236)
Median (IQR)	91 [48, 232]	96 [51, 236]	100 [54, 238]	113 [58, 270]	94 [41, 294]	90 [42, 274]	96 [45, 283]	88 [44, 221]
Total number of Radiation Oncologist with >1 visit by MA beneficiaries								
Mean (SD) [1]	----	----	----	----	----	----	----	----
Median (IQR) [1]	----	----	----	----	----	----	----	----
Total number of Radiation Oncologist with >1 visit by TM beneficiaries								
Mean (SD)	48 (48)	50 (51)	52 (52)	57 (54)	57 (60)	57 (64)	60 (68)	54 (62)
Median (IQR)	29 [15, 67]	29 [16, 72]	31 [17, 71]	37 [19, 77]	31 [14, 86]	29 [14, 78]	30 [14, 85]	27 [14, 66]

Metropolitan counties

	Regular MA plans				SNPs			
	**2016**	**2017**	**2018**	**2019**	**2016**	**2017**	**2018**	**2019**
Total number of beneficiaries with cancer under the MA plan								
Mean (SD)	704 (5196)	758 (4266)	1054 (9996)	1134 (8105)	1054 (8559)	1472 (12106)	1444 (9545)	1571 (13162)
Median (IQR)	26 [<11, 196]	27 [<11, 210]	29 [<11, 248]	24 [<11, 234]	24 [<11, 168]	28 [<11, 216]	30 [<11, 217]	28 [<11, 222]
Total number of oncology organizations with >1 visit by MA beneficiaries								
Mean (SD) [1]	----	----	----	----	----	----	----	----
Median (IQR) [1]	----	----	----	----	----	----	----	----
Total number of oncology organizations with >1 visit by TM beneficiaries								
Mean (SD)	128 (116)	131 (114)	136 (117)	141 (116)	151 (148)	148 (144)	156 (151)	142 (139)
Median (IQR)	94 [42, 176]	93 [48, 178]	99 [48, 199]	109 [51, 198]	93 [41, 216]	91 [43, 210]	98 [44, 229]	83 [42, 205]
Total number of Medical/Surgical/Hematologic/Gynecologic Oncologist with >1 visit by MA beneficiaries								
Mean (SD) [1]	----	----	----	----	----	----	----	----
Median (IQR) [1]	----	----	----	----	----	----	----	----

© 2026 Hu X, et al. *JAMA Network Open*.

Total number of Medical/Surgical/Hematologic/Gynecologic Oncologist with >1 visit by TM beneficiaries								
Mean (SD)	221 (199)	230 (206)	238 (216)	250 (223)	267 (258)	264 (259)	282 (280)	258 (263)
Median (IQR)	152 [80, 310]	157 [82, 326]	165 [83, 335]	175 [90, 347]	155 [77, 413]	155 [75, 376]	167 [78, 397]	148 [74, 353]
Total number of Radiation Oncologist with >1 visit by MA beneficiaries								
Mean (SD) [1]	----	----	----	----	----	----	----	----
Median (IQR) [1]	----	----	----	----	----	----	----	----
Total number of Radiation Oncologist with >1 visit by TM beneficiaries								
Mean (SD)	62 (51)	65 (54)	67 (57)	71 (58)	73 (64)	74 (68)	78 (73)	72 (68)
Median (IQR)	42 [24, 88]	45 [25, 94]	47 [27, 100]	52 [27, 95]	48 [23, 109]	44 [24, 110]	48 [24, 114]	47 [23, 102]
Non-metropolitan counties								
	Regular MA plans				SNPs			
	**2016**	**2017**	**2018**	**2019**	**2016**	**2017**	**2018**	**2019**
Total number of beneficiaries with cancer under the MA plan								
Mean (SD)	37 (117)	47 (146)	54 (194)	50 (171)	25 (70)	24 (53)	34 (79)	49 (227)
Median (IQR)	<11 [<11, 26]	<11 [<11, 32]	<11 [<11, 30]	<11 [<11, 26]	<11 [<11, 20]	<11 [<11, 22]	<11 [<11, 28]	<11 [<11, 33]
Total number of oncology organizations with >1 visit by MA beneficiaries								
Mean (SD) [1]	----	----	----	----	----	----	----	----
Median (IQR) [1]	----	----	----	----	----	----	----	----
Total number of oncology organizations with >1 visit by TM beneficiaries								
Mean (SD)	35 (22)	37 (24)	39 (25)	47 (39)	27 (17)	29 (19)	30 (21)	38 (40)
Median (IQR)	28 [20, 41]	31 [23, 42]	33 [23, 46]	35 [25, 54]	24 [18, 31]	25 [18, 34]	27 [20, 36]	29 [20, 38]
Total number of Medical/Surgical/Hematologic/Gynecologic Oncologist with >1 visit by MA beneficiaries								
Mean (SD) [1]	----	----	----	----	----	----	----	----
Median (IQR) [1]	----	----	----	----	----	----	----	----
Total number of Medical/Surgical/Hematologic/Gynecologic Oncologist with >1 visit by TM beneficiaries								
Mean (SD)	54 (35)	58 (38)	58 (38)	71 (68)	44 (31)	47 (36)	49 (36)	63 (73)
Median (IQR)	47 [31, 67]	50 [31, 71]	52 [31, 75]	53 [32, 83]	37 [25, 52]	39 [26, 57]	42 [26, 59]	46 [26, 69]
Total number of Radiation Oncologist with >1 visit by MA beneficiaries								
Mean (SD) [1]	----	----	----	----	----	----	----	----
Median (IQR) [1]	----	----	----	----	----	----	----	----
Total number of Radiation Oncologist with >1 visit by TM beneficiaries								
Mean (SD)	18 (12)	18 (12)	19 (12)	23 (20)	15 (10)	16 (11)	15 (11)	19 (21)
Median (IQR)	14 [<11, 22]	16 [<11, 22]	16 [11, 24]	17 [11, 27]	13 [<11, 18]	13 [<11, 20]	13 [<11, 19]	14 [<11, 21]

[1] All cell sizes <11 are suppressed to comply with the data use agreement.

© 2026 Hu X, et al. *JAMA Network Open*.

eFigure 1. Missingness of Provider Identifiers on Medicare Advantage Plan Level

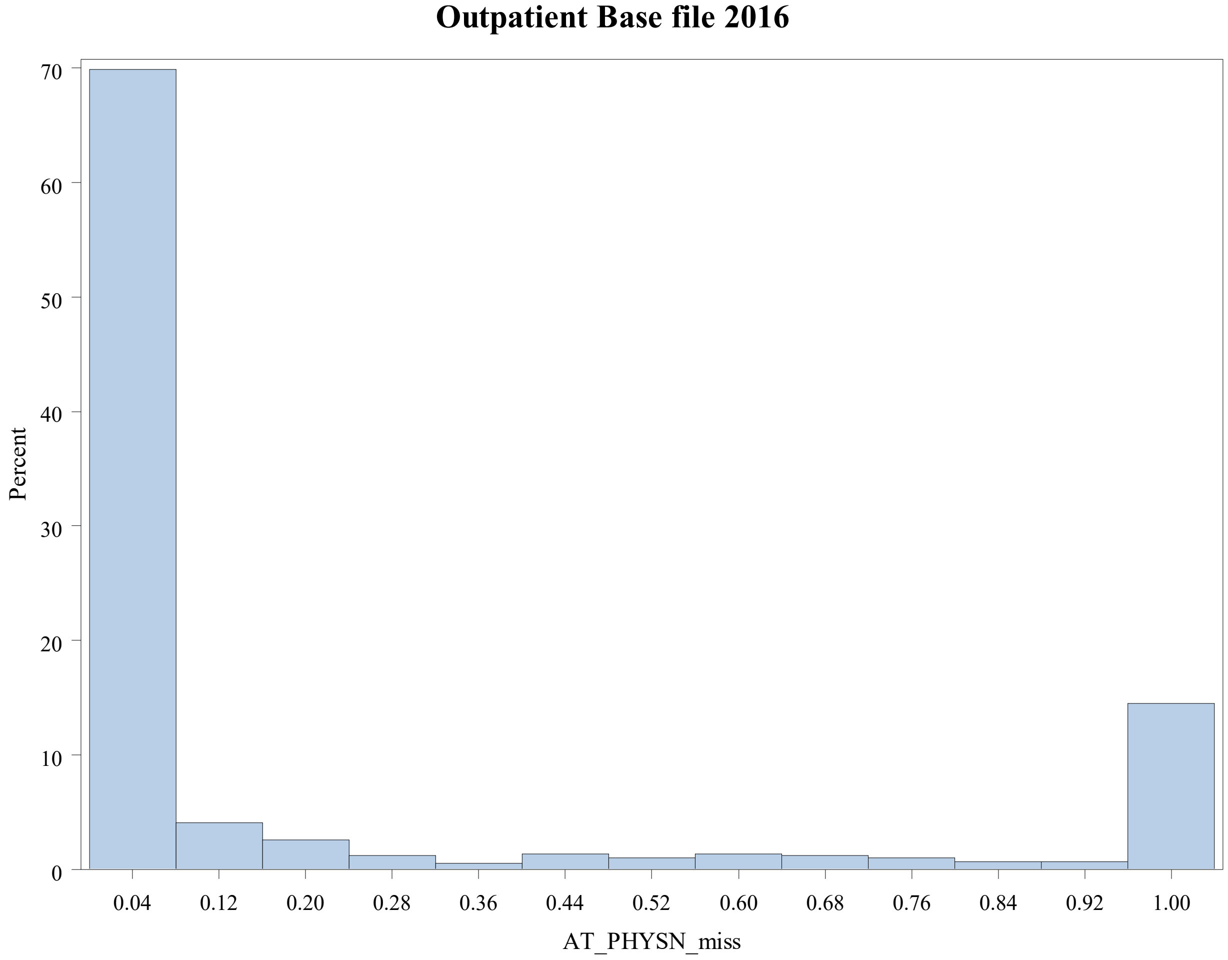

**Notes:* example using outpatient base file in 2016. Attending physician provider identifier is the most complete field and is used to examine missingness on the Medicare Advantage plan (Contract ID) level.

© 2026 Hu X, et al. *JAMA Network Open*.

eFigure 2. Sample Flow Chart

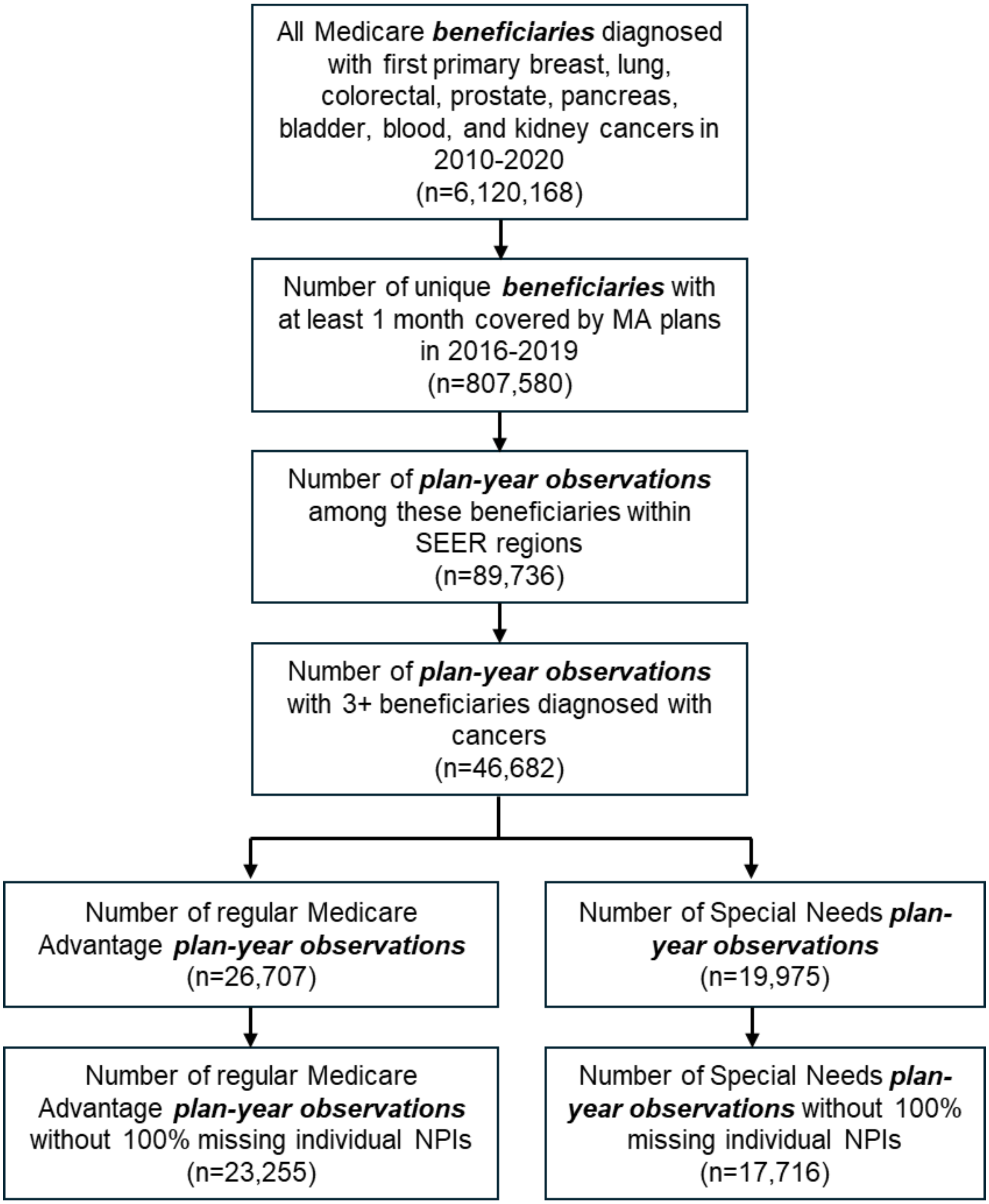

© 2026 Hu X, et al. *JAMA Network Open*.

eFigure 3. Trend of Narrow Effective Oncology Provider Network

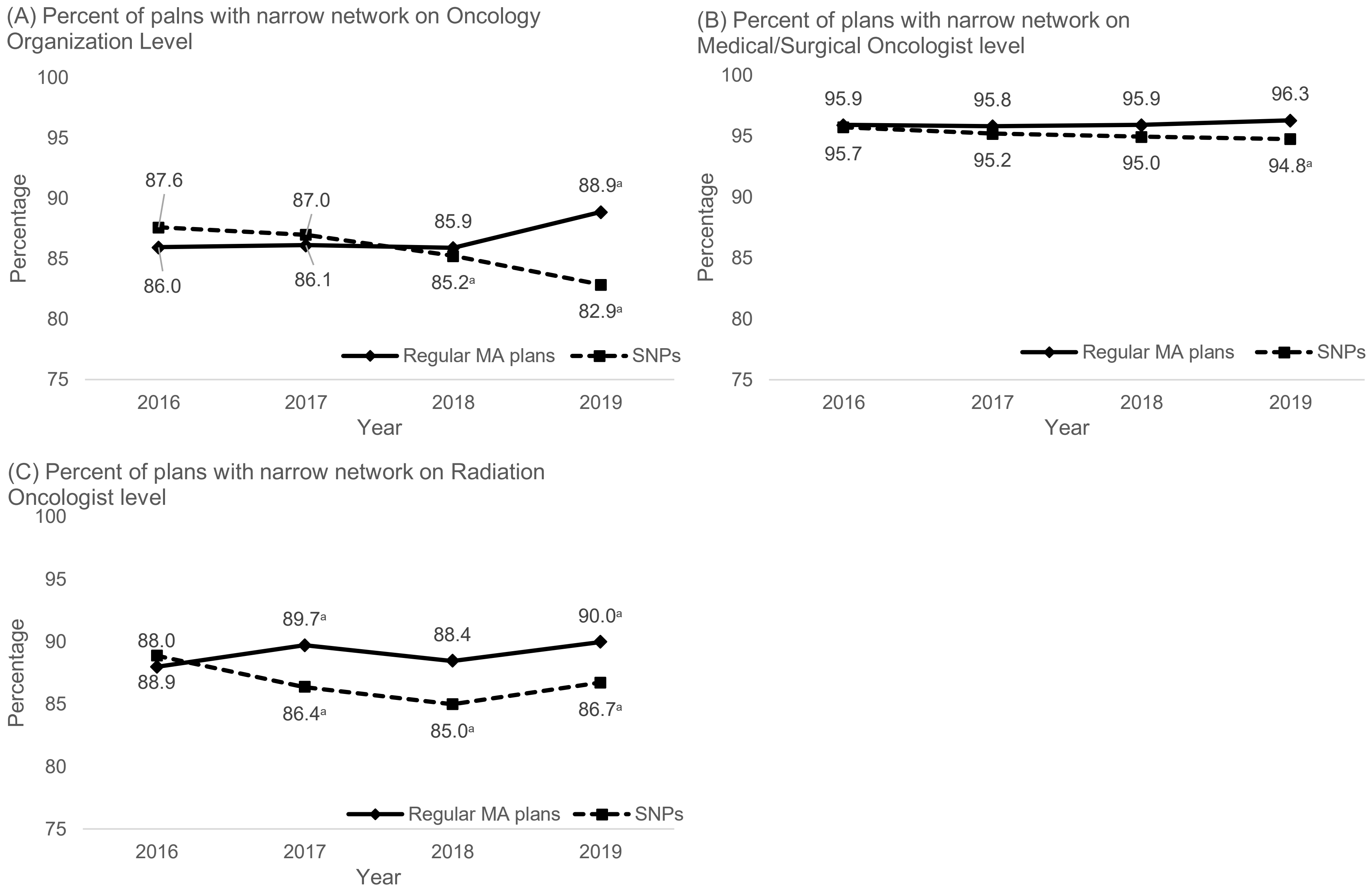

[a] *p-value* < 0.05 compared to reference year (2016).

© 2026 Hu X, et al. *JAMA Network Open*.

eFigure 4. Trend of Effective Oncology Provider Network Metrics Among Plans with <10% Missing Inpatient and/or Outpatient Visits

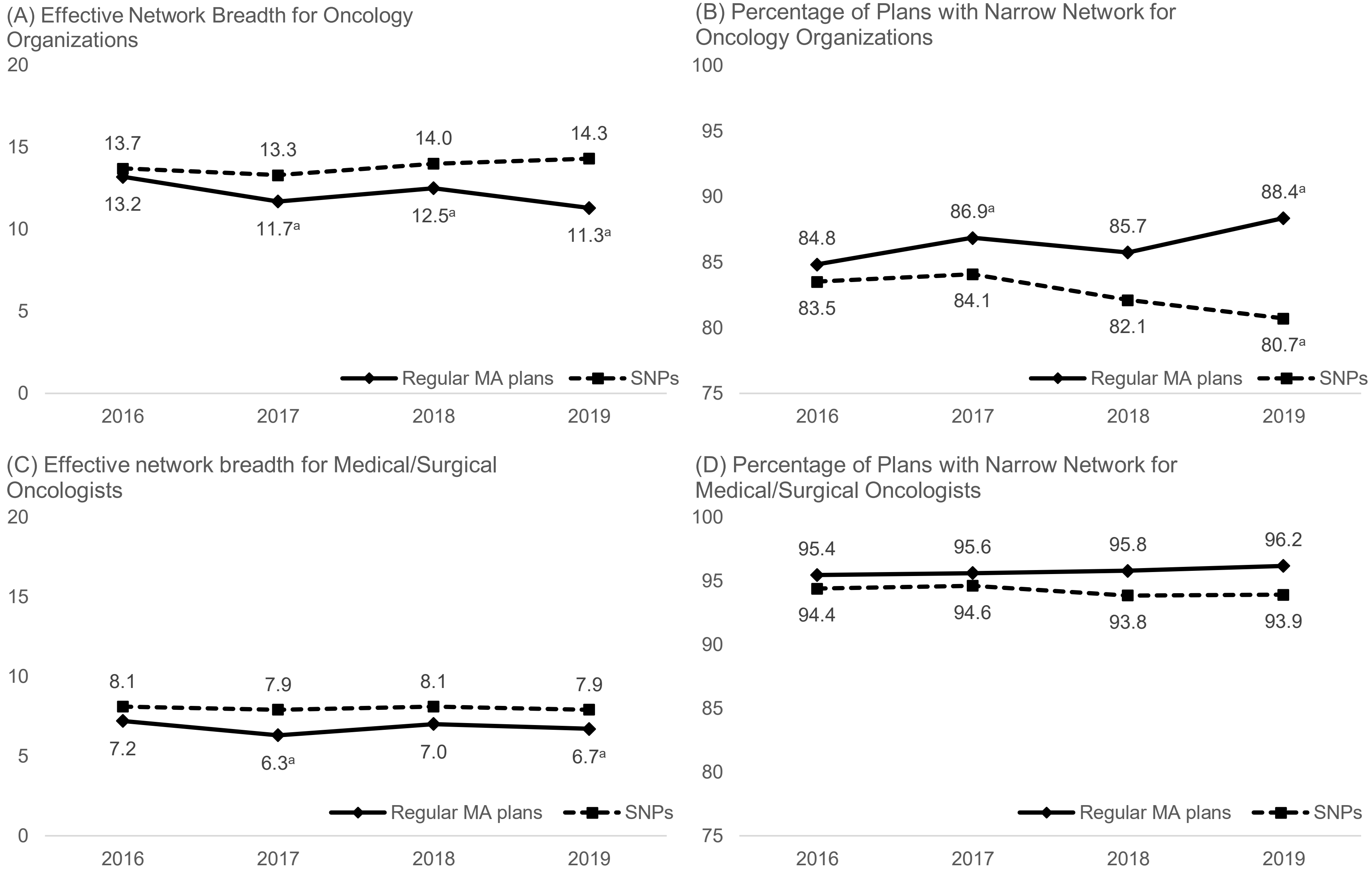

© 2026 Hu X, et al. *JAMA Network Open*.

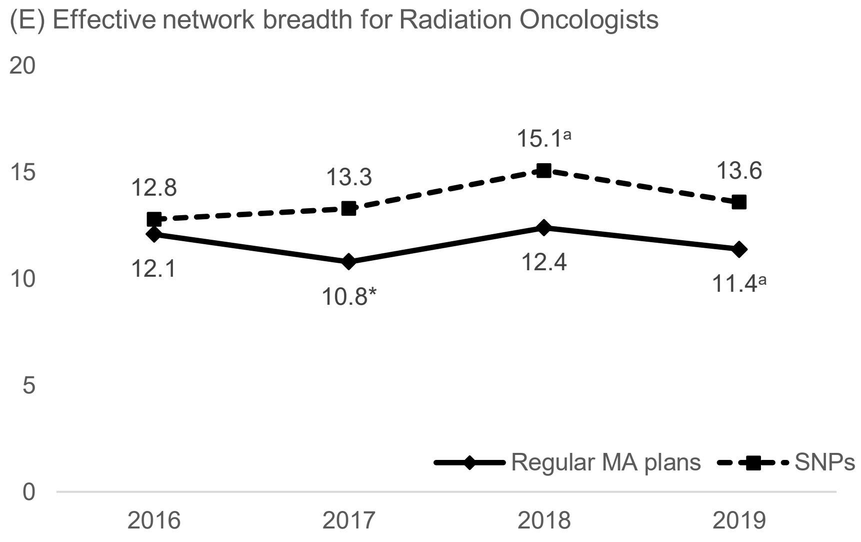

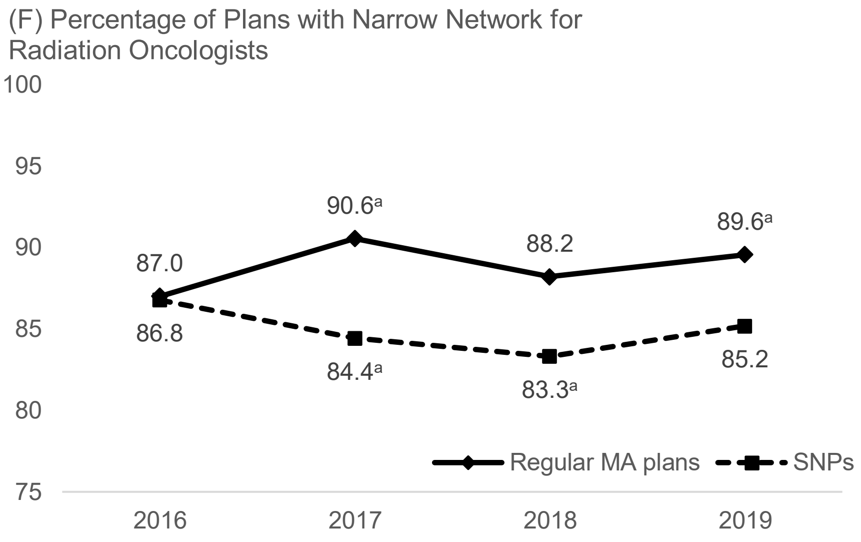

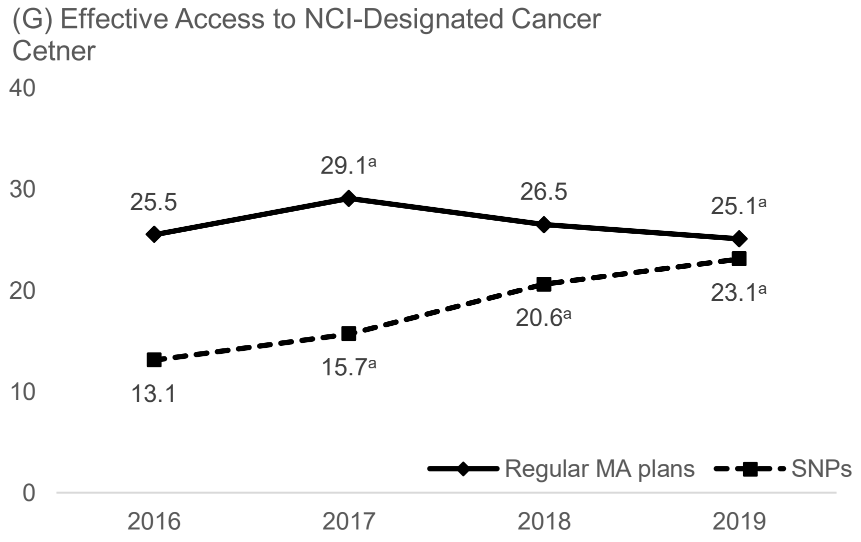

* *p-value* < 0.05 compared to reference year (2016).

© 2026 Hu X, et al. *JAMA Network Open*.

eFigure 5. Trend of Effective Oncology Provider Network Metrics Among Plans with At Least 5 Beneficiaries with Cancer

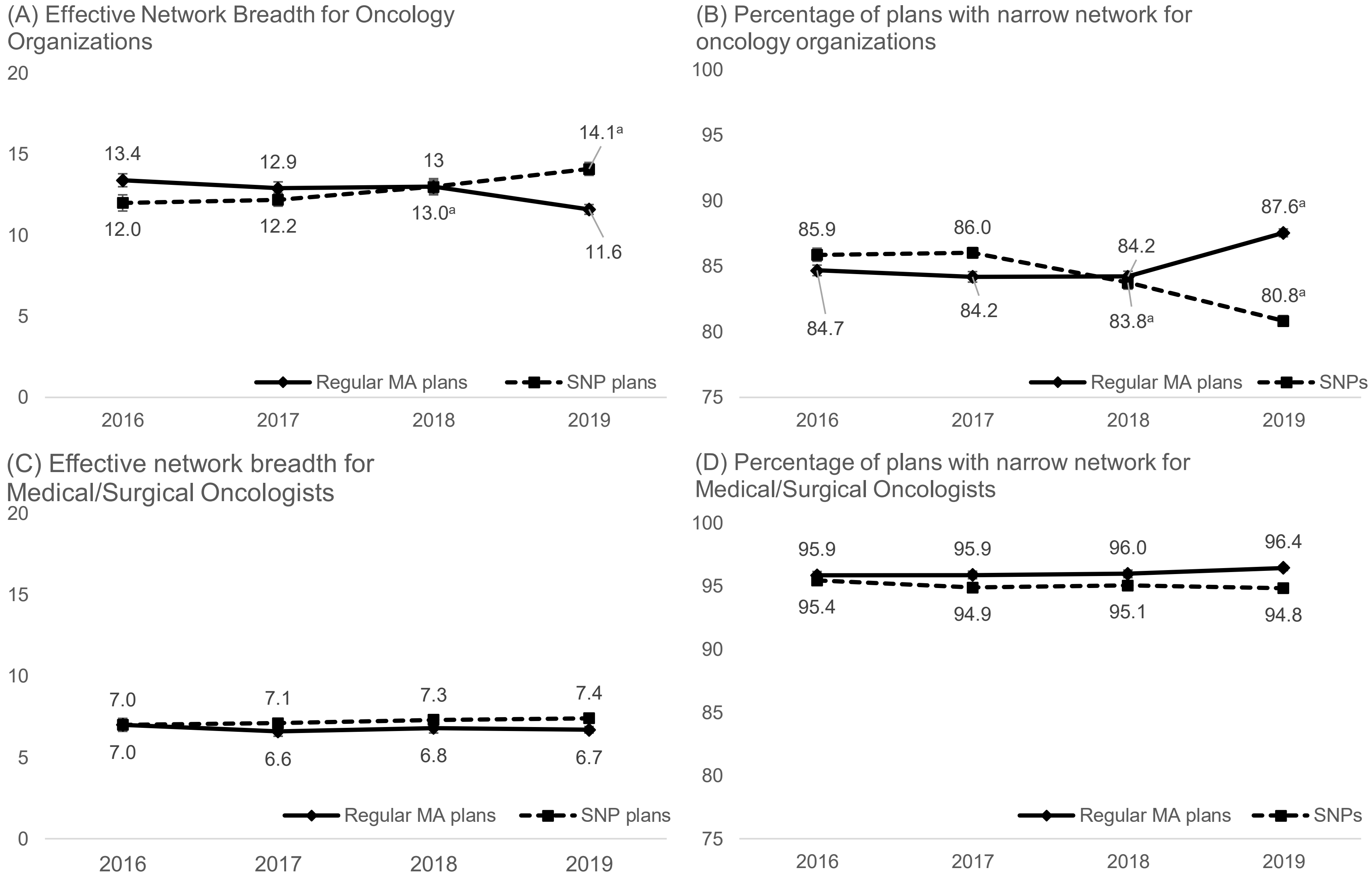

© 2026 Hu X, et al. *JAMA Network Open*.

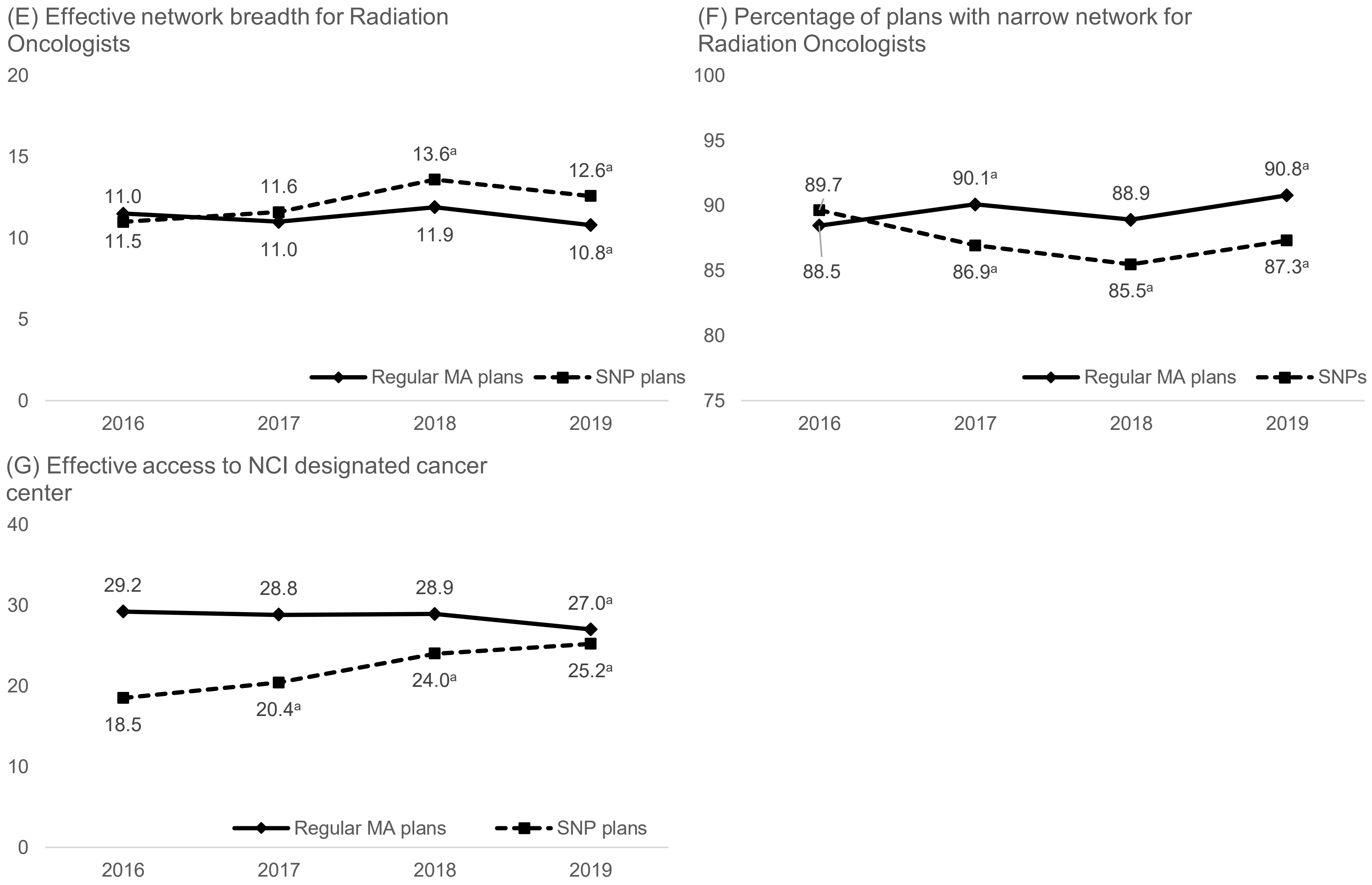

* *p-value* < 0.05 compared to reference year (2016)

© 2026 Hu X, et al. *JAMA Network Open*.

eFigure 6. Trend of Effective Oncology Provider Network Metrics Stratifying by >100 and ≤100 Beneficiaries with Cancers Under the Medicare Advantage Plans

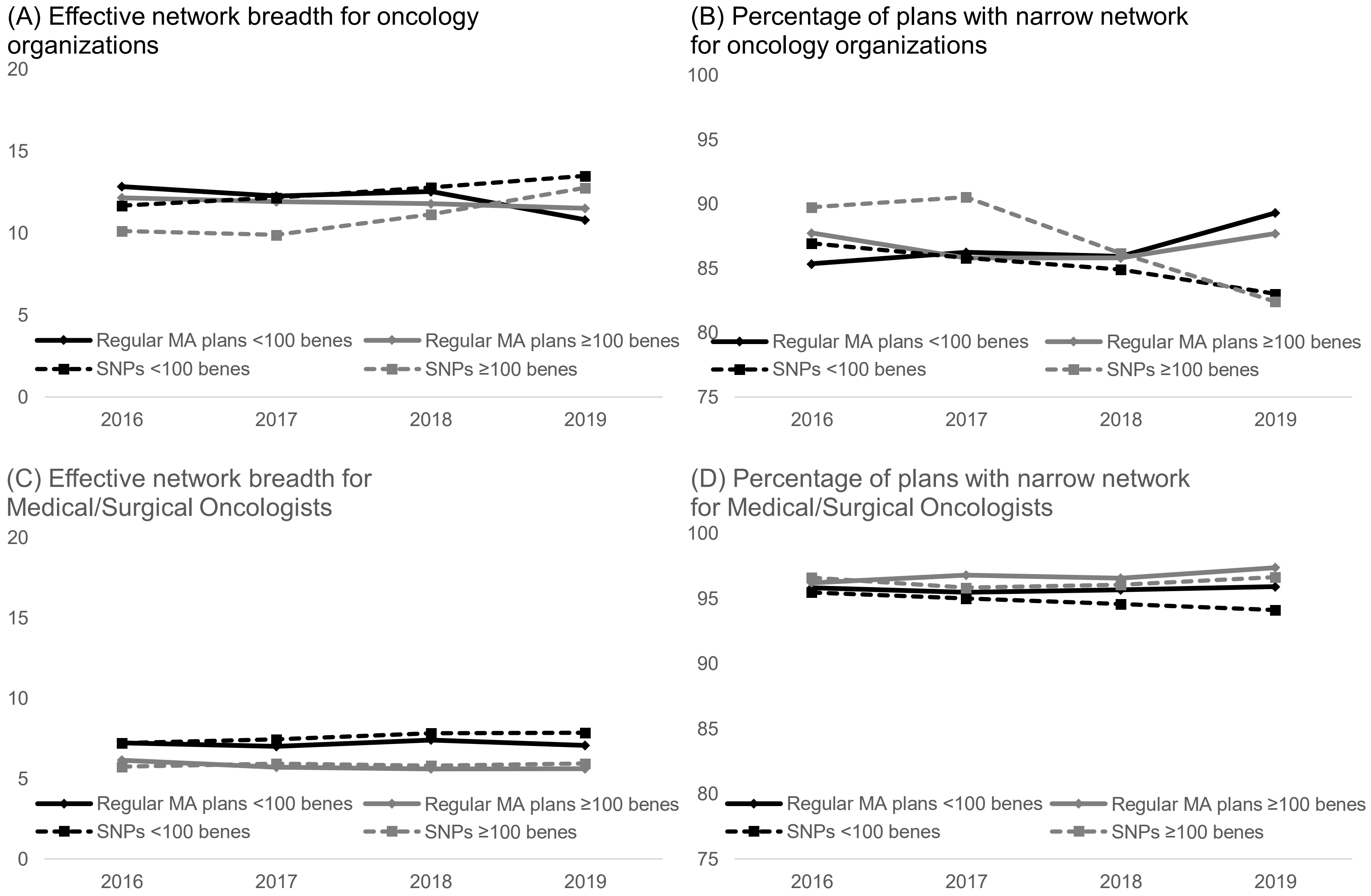

© 2026 Hu X, et al. *JAMA Network Open*.

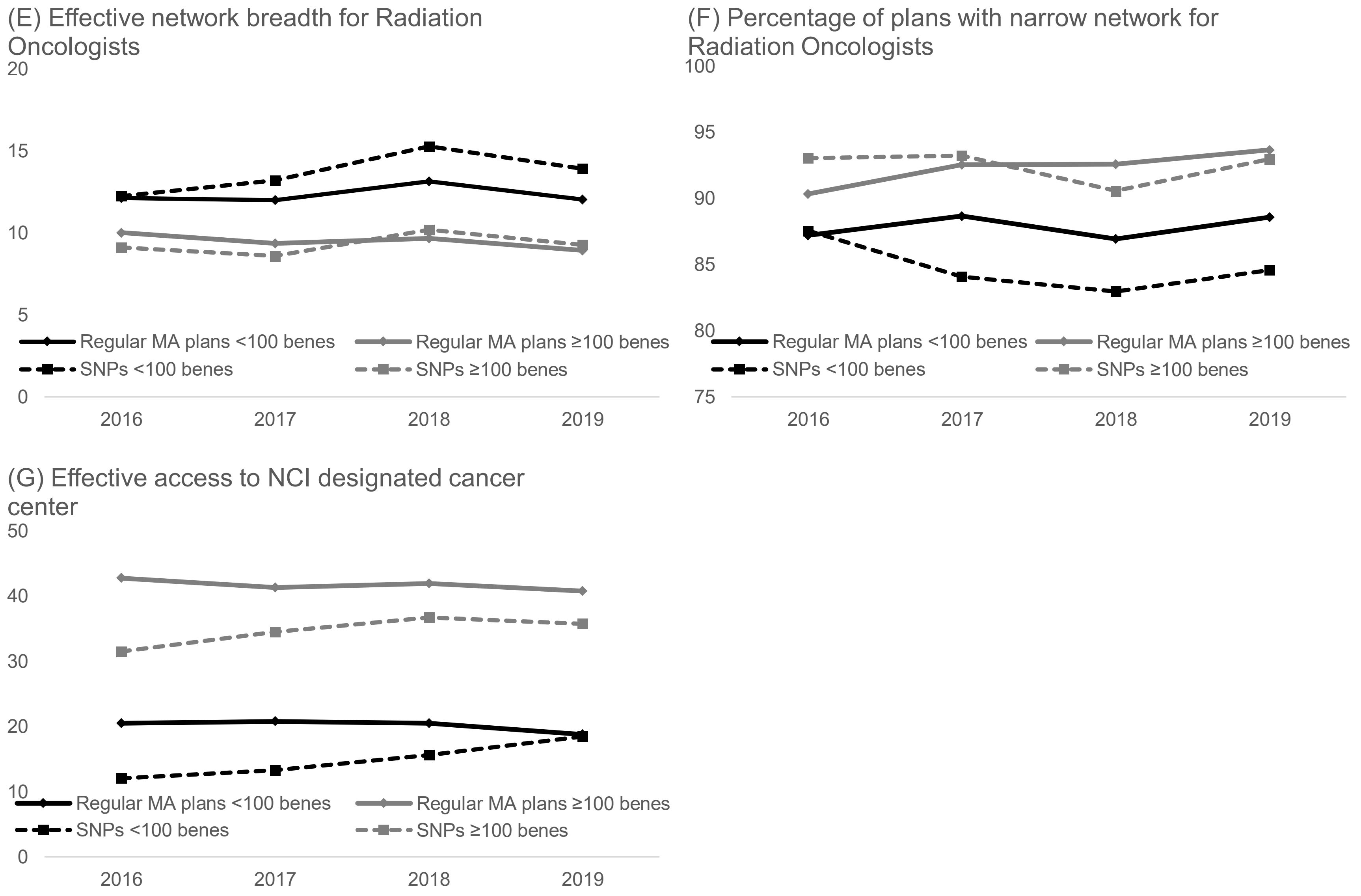

© 2026 Hu X, et al. *JAMA Network Open*.

eFigure 7. Trend of Effective Oncology Provider Network Metrics Excluding Plans in California

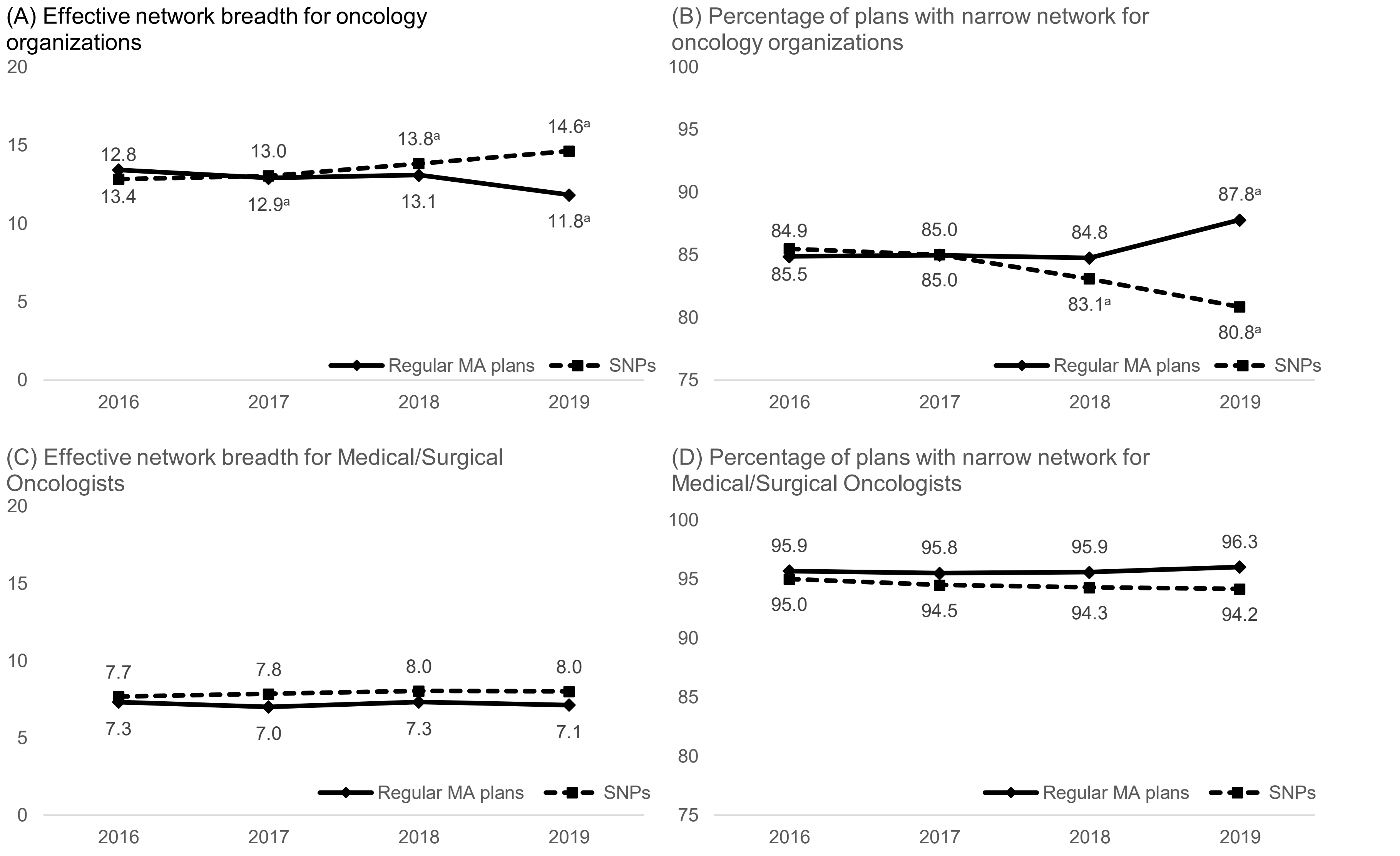

© 2026 Hu X, et al. *JAMA Network Open*.

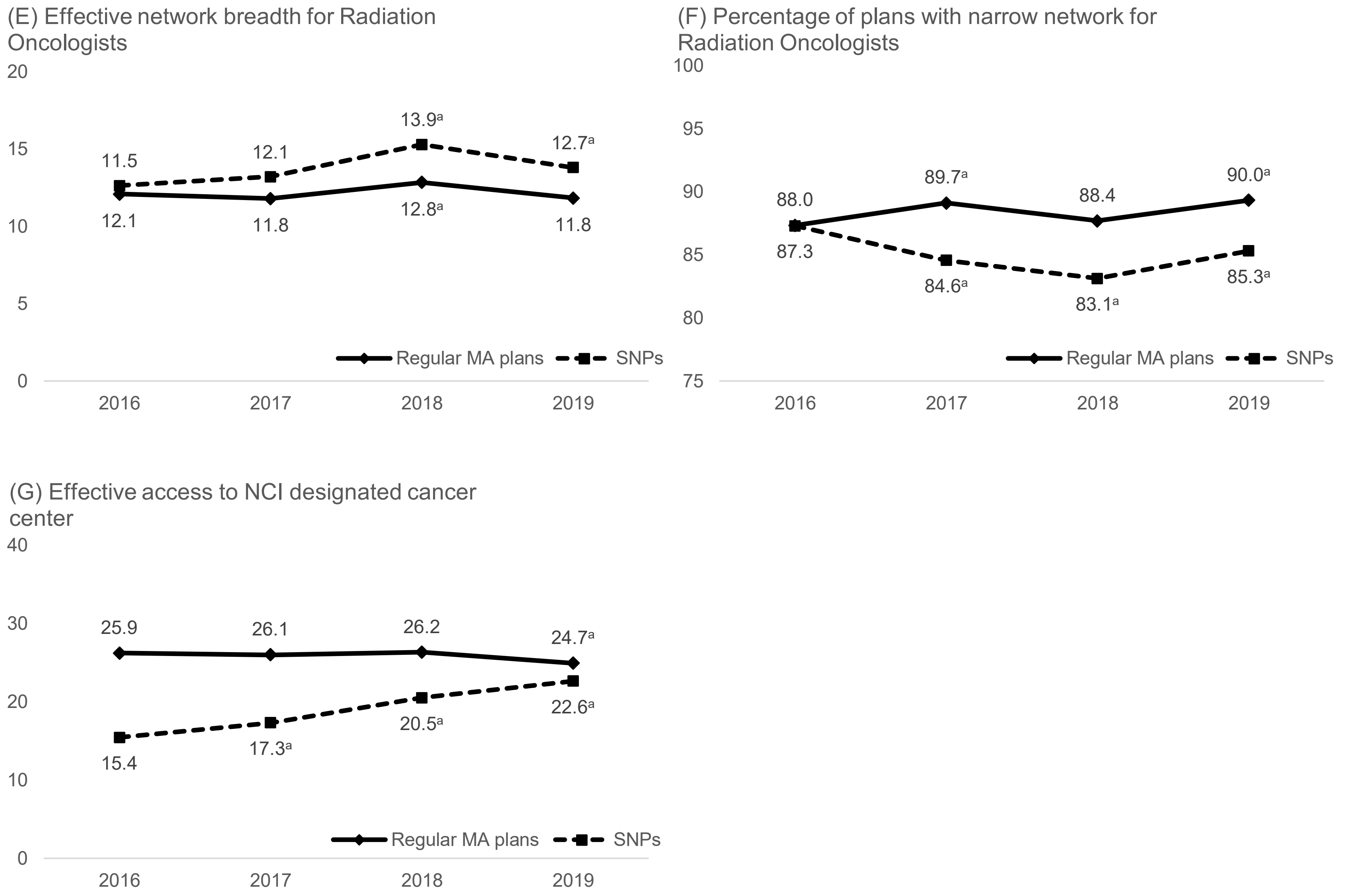

* *p-value* < 0.05 compared to reference year (2016)

© 2026 Hu X, et al. *JAMA Network Open*.
