# Supplementary material for: Oncology Organization and Oncologist Networks Under Medicare Advantage Plans
Source: JAMA Netw Open. 2026 Jun 15;9(6):e2618507. doi: 10.1001/jamanetworkopen.2026.18507 (PMC13270271; doi:10.1001/jamanetworkopen.2026.18507)
Supplement: Supplement 2. — Data Sharing Statement [file jamanetwopen-e2618507-s002.pdf]

Data Sharing Statement

Hu. Oncology Organization and Oncologist Networks Under Medicare Advantage Plans. *JAMA Netw Open*. Published June 15, 2026. doi:10.1001/jamanetworkopen.2026.18507

Data
Data available: No

Additional Information
Explanation for why data not available: The dataset used to conduct this study are available upon approval of a research protocol from the National Cancer Institute. Instructions for obtaining these data are available at https://healthcaredelivery.cancer.gov/seermedicare/obtain/.
